# Joint-Based Action Progress Prediction

**DOI:** 10.3390/s23010520

**Published:** 2023-01-03

**Authors:** Davide Pucci, Federico Becattini, Alberto Del Bimbo

**Affiliations:** 1 Media Integration and Communication Center (MICC), University of Florence, 50124 Firenze, Italy; 2 Dipartimento Di Ingegneria Dell’Informazione E Scienze Matematiche, University of Siena, 53100 Siena, Italy

**Keywords:** action progress prediction, body joints, body pose

## Abstract

Action understanding is a fundamental computer vision branch for several applications, ranging from surveillance to robotics. Most works deal with localizing and recognizing the action in both time and space, without providing a characterization of its evolution. Recent works have addressed the prediction of action progress, which is an estimate of how far the action has advanced as it is performed. In this paper, we propose to predict action progress using a different modality compared to previous methods: body joints. Human body joints carry very precise information about human poses, which we believe are a much more lightweight and effective way of characterizing actions and therefore their execution. Estimating action progress can in fact be determined based on the understanding of how key poses follow each other during the development of an activity. We show how an action progress prediction model can exploit body joints and integrate it with modules providing keypoint and action information in order to be run directly from raw pixels. The proposed method is experimentally validated on the Penn Action Dataset.

## 1. Introduction

Action recognition is an important field of research in computer vision that focuses on the problem of identifying and categorizing the actions that are being performed by individuals. This is a challenging task, as the same action can be performed in many different ways, and can be affected by factors such as the viewpoint, lighting conditions, and the presence of other objects in the scene. Despite these challenges, action recognition has many potential applications in fields such as video surveillance [1,2], sports analysis [3,4,5], and human–computer interaction [6,7,8].

There are several different techniques that can be used to perform action recognition in images or videos, exploiting different input modalities [4,9,10]. The simplest and most common method is to directly infer the action from raw frames [11]. This approach uses RGB frames as the input to a machine learning model, which is trained to identify and classify the actions that are being performed. This is a widely used approach, as it is relatively simple and can be effective for many types of actions. Other methods exploit optical flow to perform action recognition [4,12,13]. These approaches use the movement of pixels between consecutive frames in a video to determine the motion of the objects in the scene. This information can then be used to identify and classify the actions that are being performed. This approach can be more robust to factors such as viewpoint and lighting, but can be sensitive to noise.

Following a recent crop of literature [14,15,16,17,18], in this paper we are interested in recognizing the action from a pose-based point of view. Pose-based action recognition is an approach that uses information about the position and orientation of human body joints to identify and classify actions. This approach is based on the idea that the movements of body joints are a key element of many actions, and that by analyzing the positions of these joints over time, it is possible to accurately identify and classify the actions that are being performed. To perform action recognition using body joints, the most common approach is to use a convolutional neural network (CNN) to process frames and identify the key features or elements that are relevant to the body joints in question [19,20]. This information can then be used to train a model that can make predictions about the likely actions that are being performed in a given image or video.

Regardless of data modality, action recognition can be declined under several aspects. The action must be recognized, but it must also be localized in time [21,22] and space [23,24,25]. In addition, one recent development in the field of action recognition is the ability to predict the progress of the ongoing action [26,27]. This allows to not only identify the action that is being performed, but also to make predictions about how the action is evolving over time. It has many potential applications, such as in the fields of medicine and sports, where being able to predict the progress of an action can help to identify potential problems or injuries, and allow for early intervention [27]. Additionally, this ability to predict the progress of an action can also be useful in other areas, such as video surveillance and video summarization, where it can help to identify key events and highlight important information for the user.

In this paper, we propose to exploit a pose-based approach for action progress prediction. By leveraging body joints we are able to train a lightweight and efficient model that is able to estimate how far an observed action has progressed. The usage of body joints rather than RGB pixels allows the model to analyze sequences of poses which compose an action, thus allowing it to identify relevant motion patterns that correlate with the development of the action itself. We complement our model with an action classification module to understand which action is being performed and we show the effect of using predicted body joints rather than accessing a source of joints (e.g., a 3D sensor such as a Kinect [28]). The main contributions of our paper are the following:

- We present a joint-based action prediction model. The architecture is based on a recurrent model that estimates the progress of the observed action as it is performed, emitting predictions online, for every frame. To the best of our knowledge, we are the first to adopt a joint-based approach for action progress prediction.
- We add to our progress prediction model additional modules to estimate body joints and the category of the ongoing action. This allows us to estimate progress directly from raw RGB pixels, reasoning on joint positions.
- The proposed progress prediction model is highly efficient and can be used in real-time online settings. We propose an analysis of the execution cost under different scenarios, depending on different degrees of data availability.

## 2. Related Work

Action recognition is an extensively studied area in computer vision [13,29,30,31]. Traditionally, action recognition methods have dealt with simply classifying still images [32] or video clips [13]. In [33], the authors use action banks—large sets of individual, viewpoint-tuned action detectors. The method exploits the fact that a large number of small action detectors, when pooled together, can yield better results than traditional low-level handcrafted features in discriminating videos.

Fully understanding actions in videos however requires us to solve more complex tasks such as temporal localization and spatial detection. Temporal action localization attempts to identify video segments in which certain actions take place [34,35]. This is important to process untrimmed, arbitrarily long, videos. On the other hand, methods for action detection yield spatial locations in the form of bounding boxes [23]. Recent works have addressed both tasks simultaneously by generating spatio-temporal action tubes [24,30,36] or frame coordinates [37].

In general, a noteworthy approach for action recognition is to process two separate sources of data: RGB frames and optical flow. This approach, first presented in [13] and dubbed the Two-Stream Convolutional Network, adopts two separate CNNs to deal with the two input modalities and then applies a late fusion strategy to blend the predictions. This approach has then been used in several applications, also for spatiotemporal action detection [24,36]. In particular, [36] uses a two-stream network to process untrimmed videos and, thanks to a detection head inspired by Fast-RCNN [38], is able to generate framewise-detections, which are then linked in time with a Hungarian algorithm. Singh et al. [24] adopt a similar approach, but improve the data association module by linking detections online and using a single stage action detector [39] to work in real-time.

Another approach which is commonly used for action recognition is to exploit 3D convolutions [40,41,42]. The 3D convolutions consist of using convolutional filters that can also span over the temporal dimension in addition to the traditional spatial ones. This allows us to take into account multiple frames by processing video chunks as individual samples.

A further characterization of the evolution of an action has been recently proposed in [26], where the authors learn to predict its ongoing progress, as the action is observed. To do so, a CNN backbone extracts features of the whole frame and, thanks to a spatio-temporal detection module, feeds roi-pooled crops to a temporal model that emits the percentage of progress at every timestep. This opens up to interaction applications and has found usage also in surgical methods [27,43]. Similarly, [25] have estimated progress values by proposing a cycle-consistency learning strategy: by aligning similar frames of different videos in an embedding space, they are able to infer progress values of action subphases.

All methods that have dealt with estimating action progress, however, have disregarded the notion of pose in modeling the action. We argue that an action can be interpreted as a sequence of body poses, which can better convey progress cues rather than raw pixels. Therefore, in this work we propose to address the problem of action progress prediction from a body pose perspective, feeding our model with 2D body joints, directly inferred from the frames. A large crop of literature has studied the problem of estimating body joints from videos and single images [15,20,44,45]. One of the first approaches to do so, was a declination of Mask-RCNN, which added a joint regression head to its instance segmentation backbone [44]. Noteworthy methods from the state-of-the-art such as UniPose [20], OpenPose [45] or Alpha-Pose [46]. In this work we adopt UniPose [20] as a source for extracting body joints and train our model to estimate action progress. The model exploits Waterfall Atrous Spatial Pooling [47], which provides multiscale processing and network efficiency, in order to output heatmaps for each joint.

In our work, we combine several different modules to be able to estimate action progress directly from the pixels. Recently, a research trend known as Automated Machine Learning (AutoML) has been intensively studied to automate the processes of finding an optimal architecture for a given problem [48,49]. AutoML has given remarkable results in challenging fields such the medical one [50] and has been also successfully adopted for action recognition [51]. This could be an interesting starting point for future developments to further improve our proposed approach.

## 3. Joint-Based Action Progress Prediction

In this paper we propose a joint-based model to address the task of action progress prediction. The labels for action progress were generated To define the progress of an action, we follow the work of Becattini et al. [26], in which a linear definition is given. The same formulation has been also adopted in [25] and it defines the current progress $p_{t}$ of an action as:

(1) $$p_{t}=\frac{t-S}{E-S}\in \left[0,1\right]$$

where *t* is the current frame, *S* is the frame in which the action begins and *E* is the frame where the action ends.

**Figure 1:**
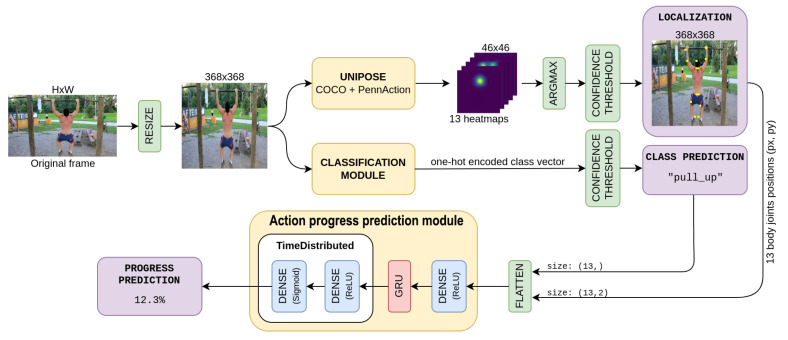
Proposed architecture for localization, action classification, and progress estimation. Blocks in purple are the outputs produced.

Our proposed approach leverages skeleton joint information to model the progression of an action as a progression of human poses. We combine several modules, in charge of inferring the pose, classifying the action and, finally, estimating action progress. The model architecture is shown in Figure 1. In the following, we first define an oracle model, capable of accessing precise pose and action class labels and we then present a fully functional model which estimates pose, category, and progress directly from raw video sequences.

### 3.1. Action Progress Prediction

The module for action progress estimation is given sequences of joints positions to predict the progress of the action. Architectures specifically designed to deal with sequences are Recurrent Neural Networks [52], which are able to memorize information progressively with the input sequence. As a result the outputs will be influenced by all previous observations.

In our work, we adopt a declination of RNN called Gated Recurrent Units (GRUs) [53]. GRUs are a simplified variant of Long Short-Term Memories (LSTM) [54]. A GRU has a hidden state acting as a memory, which gets updated at every timestep and two gates, regulating which information to store. A reset gate controls the flow of information from the previous time step, while an update gate controls the flow of information from the current input. This makes it possible to decide how much information to retain from previous time steps or how much new information to store when updating the internal state.

The update gate is typically implemented using a sigmoid activation function, while the reset gate is typically implemented using a tanh activation function. The output of the update and reset gates is multiplied element-wise with the input and the hidden state, respectively, and the resulting values are used to compute the new hidden state for the current time step.

One advantage of GRUs over LSTMs is that they have fewer parameters, which makes them faster to train and easier to optimize. They also tend to perform well on a wide range of tasks, including language modeling, machine translation, and speech recognition. Dense layers are employed before the GRU stage, in order to learn a feature representation for joints positions. A final dense layer is also used in order to produce the progress percentage.

As input to the model we also assume to have access to a class label for the observed action. In practice, this can be estimated by an action classifier, as detailed in Section 3.3. When dealing with trimmed actions, each action starts end ends with the video. The starting point *S* of the action is always found in the first frame and the ending point *E* in the last one. In real applications, *S* can be estimated by the action classifier, which signals the model to start estimating the progress only when an action is detected. In a similar fashion, *E* can be estimated by the action classifier once no-action is detected, or heuristically when the model understands that the action is at its end (e.g., progress above a threshold). In such a way, it is possible to understand *when* an action takes place in a video and what its *progress* is, including in cyclic scenarios (e.g., multiple push-ups). As we assume the action classification is already available at the time the action progress model is executed, we add as input the one-hot encoded class to the model.

**Figure 2:**
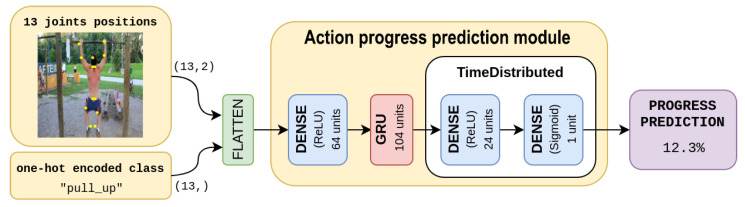
Action Progress Prediction architecture.

Our action progress prediction model is depicted in detail in Figure 2: the first dense stage builds a feature representation of the input, then the GRU stage allows a degree of memorization which is fundamental for the task, finally, the two time-distributed dense layers produce the progress prediction for each frame.

We study two possible paradigms that can be used in our task:

- *Many-to-One*: we consider a window of *N* frames that are passed sequentially to the network. After all such frames have been considered, a single output is produced, that is the progress prediction for the last frame in the input sequence. When a new frame is available, the window is moved in order to consider the *N* most recent frames.
- *Many-to-Many*: each frame of the sequence is passed in real-time to the network, producing the progress prediction for that frame. The network has an internal state which is maintained and updated throughout the whole sequence so that previous frames influence the prediction relative to the current frame.

We feed to the model normalized joints with coordinates scaled in [0, 1], dividing coordinates by the size of the frame.

To train the model we used the Adam Optimizer [55] with a learning rate $\alpha =5\;\times \;10^{-2}$, and batches of 16 videos. The loss employed is mean absolute error (MAE). During training, data augmentation was applied, performing a random rotation of joints positions in $\left[-10^{\circ },+10^{\circ }\right]$ and a horizontal flip with p=0.5.

### 3.2. Joint Extraction—UniPose

**Figure 3:**
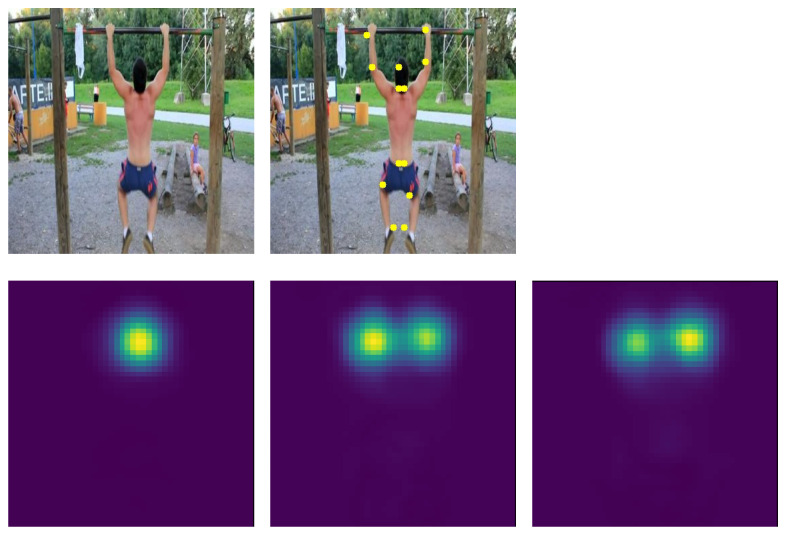
Output of UniPose when an example image is processed. Below are available (**left** to **right**) the heatmaps produced for the head position, the **left elbow** and the **right elbow**.

In order to test our model directly on videos, we need a module for body-joints extraction. We use the state-of-the-art body-joints extractor UniPose, by Artacho et al. [20]. This model makes use of ResNet [56] as a backbone module, followed by a Waterfall Atrous Spatial Pooling [47]. Given a frame, the output of UniPose consists in K heatmaps, each associated to a distinct joint. A peak in a heatmap constitutes the location prediction for the corresponding joint (an example is shown in Figure 3).

We use the UniPose model pretrained on the COCO dataset [57], which predicts the position of 17 key-points of the human body. We then finetune UniPose performing 50 additional training epochs. Once again, the Adam optimizer is used, with a learning rate of $1\;\times \;10^{-5}$ and a batch size of 8. Data augmentation techniques were employed during the training, applying a random rotation of videos in $\left[-10^{\circ },+10^{\circ }\right]$ and a horizontal flip with p=0.5.

The UniPose model is then connected to the progress estimation module in order to process videos directly from raw pixels rather than an oracle source of joints. A confidence threshold of 0.5 is applied to the predictions of UniPose, so that the model is given only trustworthy joints. The ones that do not respect the threshold are placed in (0,0) thus ignored for progress estimation. In order to enhance the performances when dealing with joints extracted by UniPose, a fine-tuning of the progress prediction module is performed, training further for 50 epochs. Adam optimizer is used with a learning rate of $1\;\times \;10^{-4}$. The same joint data augmentation is applied, as outlined in Section 3.1.

### 3.3. Classification Module

In order to feed classification labels to the network, a classifier is needed to estimate *when* an action takes place in a video and *what* action is being performed. The classification module needs to estimate if an action is being performed in the current frame and, if so, to detect which action is executed. This behavior can be achieved with different strategies.

A first idea could be to decompose the module into two classifiers: a binary classifier able to distinguish among action and no-action, followed by a multi-class classifier able to detect the class of the action being performed, which is activated only if the first classifier detects an action. Another idea could be to have a single multi-class classifier in which a no-action class is available together with all the possible action classes.

In order to train such models, frames labeled as no-action need to be available in the training set. As a simple solution, we distinguish among action and no-action by looking at the confidence of the prediction of the action classifier: we can consider that no action is performed when the confidence for a prediction is below a fixed threshold.

Several architectures were tested in order to obtain the best performances over this classification task. In particular, two types of backbones were tested: VGG16 [58] and InceptionV3 [59]. As we are dealing with videos, we also tested a fine-tuned architecture containing a GRU layer.

We trained the various configurations of the classifier making use of the Adam Optimizer [55] and Categorical Cross-Entropy Loss. Data augmentation techniques were employed during training, applying a random rotation of videos in [−π/10,+π/10], a horizontal flip with p=0.5, a random zoom with 0.2 as maximum factor, and other image manipulations: random brightness, saturation, hue, and contrast.

The classifier is then completed, adding a confidence threshold at 0.9 in order to distinguish among action and no-action. Finally, this module can be added to the overall architecture which is reported in Figure 1.

## 4. Dataset

**Figure 4:**
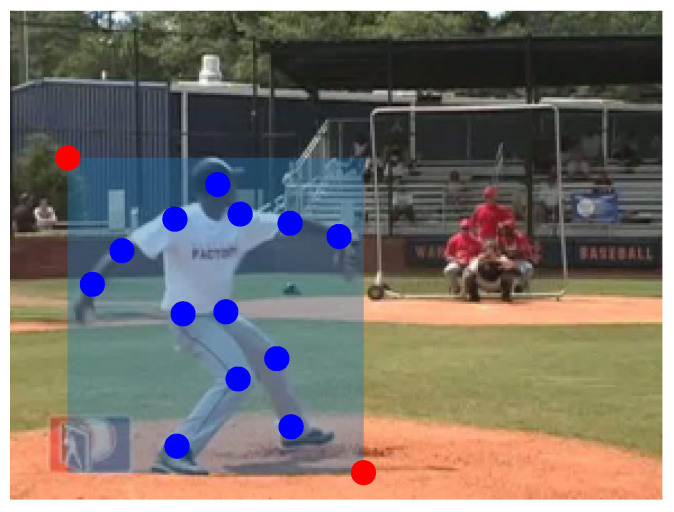
Example of annotated frame in Penn Action dataset.

The dataset used to train and test each module of the architecture is Penn Action dataset [60], which consists of 2326 video sequences of 15 different human actions. Each frame in the dataset is annotated with 13 human joints positions and their visibility, together with bounding-box position. An example of such annotations can be seen in Figure 4.

As our goal is to train a model able to estimate the progress of an action. We discarded two classes of videos that were not suitable (jump_rope and strumming_guitar), as both the starting and ending of the action are hard to identify due to its cyclic nature. All other videos were already trimmed to precisely show one single action in its entirety, without repetitions.

**Figure 5:**
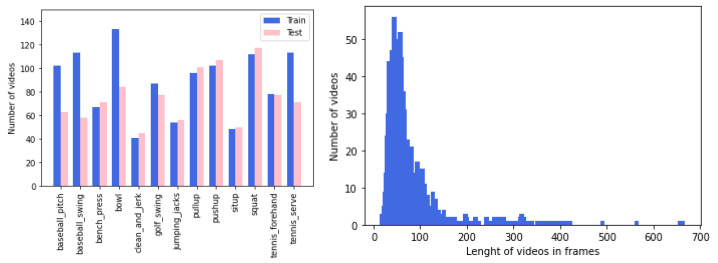
(**left**) Number of videos per class in train and test sets; (**right**) length distribution of Penn Action videos in frames.

We focused on the remaining 13 classes, for a total of 2150 videos. The train/test splits of the original dataset are used, with 1172 videos for the training set and 978 for the test set. Among the 1172 train videos, 26 (2 per each class) were selected for validation purposes. In Figure 5 (left), more details are available about the video distribution over the 13 classes in train and test sets. The videos are on average 70 frames long and are distributed as shown in Figure 5 (right).

This dataset is well suited for our task, as we are given labels to perform a supervised training for joints extraction and action classification. Most importantly, we can define progress estimation labels as a linear definition for action progress can be used, in a similar fashion to what was used in Becattini et al. [26]. This dataset was used previously used in [25], proving its appropriateness with action progress related tasks. Other datasets used in prior works such as UCF-101 [61] do not have annotated joints and therefore are not suitable for our work.

## 5. Experiments

In this section we experimentally validate our joint-based approach for action progress prediction on the Penn Action Dataset. We first present the results for the oracle model, which directly receives ground truth joint positions and action labels and we then evaluate our model by adding the joint extraction and action classification modules. We also provide an inference time analysis.

### 5.1. Oracle Model

In Table 1, we report the configurations tested for the oracle model, together with their performances over the test set. The evaluation metric here is Mean Absolute Error (MAE) as it allows good interpretability for this task. We test the two inference configurations, many-to-one and many-to-many, as detailed in Section 3.1. Among the two, the many-to-many model proved to yield the best results. We attribute this to the ability to keep track of the ongoing action in its entirety, starting from the beginning and up to the current point. On the other hand, the many-to-one approach only observes a fixed sliding window of frames. We also trained the model by changing the number of GRU stages by stacking two layers on top of each other. Interestingly, using two stages leads the model to overfit, thus slightly rising the mean absolute error on the test set. Overall, the best configuration resulted in a many-to-many configuration with a single GRU stage, with a mean absolute error on the test set of 6.22%. As a reference, we show also a variant of the model without the class data as input. This version only takes joints into account, having to understand the ongoing action as well as its development. This leads to an increase in almost 2% in the error.

From a computational point of view, the trained model turns out to be extremely fast thanks to its simplicity. Table 2 reports the average execution times for our model to process all the 72,342 frames of the 978 test videos, when running in CPU and in GPU. To benchmark our model, we used an NVIDIA Tesla T4 GPU and an Intel Xeon CPU@2.20GHz.

### 5.2. Progress Prediction with Body Joints Estimation

In order to make the model usable in a real application, we need to infer body joints from RGB frames. Here, we show the results of our model when replacing the joints oracle with Unipose, as explained in Section 3.2. We use a Unipose model pretrained on COCO [57], which is able to estimate 17 body joints, including the 13 keypoints used by the Penn Action dataset. To obtain joints similar to the ones used to train our action prediction module we simply discard the points not annotated in Penn Action. Unipose is then finetuned on the action dataset to generate only joints of interest. We evaluate the quality of the estimated body joints using the Percentage of Correct Key-points (PCK) metric, in particular PCK@0.2. This metric considers the prediction of a key-point as correct when the distance between a joint detection and the ground truth is below 20% of the torso diameter. The resulting model has a PCK@0.2 of 86.94% on the test set, which is obtained taking into account also occluded and out-of-the frame joints.

Once the joint extractor is well trained, we perform an additional finetuning step of the progress estimation module, in order to make it more robust to noisy joints. Table 3 shows the performances of the architecture when the two tuning phases are applied. The evaluation metric considered is once again Mean Absolute Error (MAE), which is evaluated over the test set of the Penn Action dataset. In this setup, the model is also given as input the ground truth class labels of each video. It is clear from the results that the two tuning phases allow a great boost in performances, proving the architecture is suitable for the task and is well trained. The table also highlights the slight drop in performances when moving from ground truth joints on to extracted ones.

The 72,342 frames of the 978 test videos were processed at a frame-rate of around 30 FPS on an NVIDIA Tesla T4 GPU. The architecture is much slower now due to UniPose, but can still run in real-time.

### 5.3. Progress Prediction with Action Classifier

**Figure 6:**
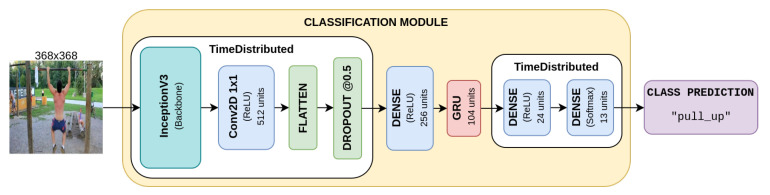
Proposed architecture for classification based on InceptionV3 backbone.

We complete the evaluation of our model by including an action classifier, instead of directly feeding action category labels to the progress prediction module. We tested several architectures for classification, as explained in Section 3.3. Table 4 reports the training details of the tested architectures, together with the performance achieved over the test set in terms of accuracy. As we can see the InceptionV3 [59] backbone achieves the best results compared to a VGG16 [58] backbone. We test the InceptionV3 model in different settings. We either finetune a pretrained model in Imagenet or we use it to extract features and just learn an action classifier to categorize them. Finetuning the whole model leads to an improvement, although not so significant. On the other hand, a considerable gain is obtained when including a GRU layer right before the dense output stage. This model accumulates information over time and emits a prediction at every timestep. Temporal modeling appears to be highly important for this type of task. To compact the features extracted from InceptionV3, we apply a 2D convolution after the last convolutional layer of the model and then we flatten and squeeze to a 256-dimensional vector the resulting feature map. The overall action classification architecture is depicted in Figure 6.

This InceptionV3-based architecture proved to be fast enough to run in real time. On average, to process 15 consecutive frames it achieves a throughput of approximately 112 FPS on an NVIDIA Tesla T4 GPU.

UniPose and the Classification Module could work in parallel, as their task is completely independent. As a result the whole architecture can run at about 30 FPS as UniPose is the slowest between the two. Although, even running the two models in series, the overall throughput is ${\left({\left(30\;\mathrm{FPS}\right)}^{-1}+{\left(112\;\mathrm{FPS}\right)}^{-1}\right)}^{-1}\approx 23\;\mathrm{FPS}$.

### 5.4. Results

**Figure 7:**
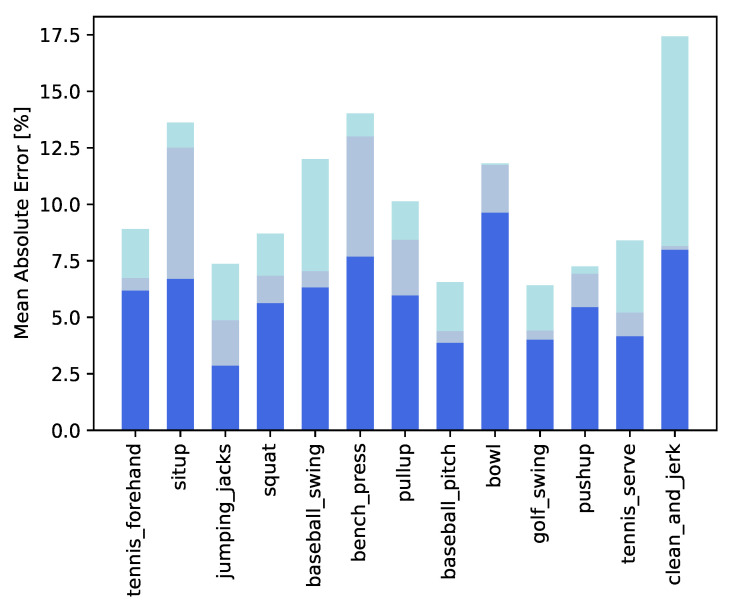
Per-class Mean Absolute Errors when using ground truth joints and ground truth class labels (in blue), when using extracted joints and ground truth class labels (in steelblue) and when everything is extracted by the architecture (in lightblue).

Here we test the overall architecture, highlighting the impact of every module in the final action progress results. Once again the evaluation metric for the model is Mean Absolute Error (MAE), evaluated over Penn Action dataset test set. Figure 7 reports a per-class comparison in terms of MAE when both joints and class labels are estimated by the architecture. For body joints we use UniPose, as explained in Section 3.2 and for class labels we use the action classifier presented in Section 3.3. Figure 7 also reports the results obtained by the model when either the joints or the action label are given. Interestingly, when estimating joints using UniPose, the classes of bench_press and situp become much more challenging. We impute this to a higher level of body self-occlusion, which makes it harder for UniPose to estimate the joints correctly.

When introducing also the action classifier, there is a small increase in MAE distributed over all classes with the notable exception of clean_and_jerk, which results much more problematic. Looking back at Figure 5 we can notice that only 40 videos are available in the train set for clean_and_jerk, hence the training of the classifier results harder for this class compared to the others. To limit the effect of the data unbalance, during training we build a batch by selecting at random a video per class. This slightly helps the model, yielding an MSE of 0.1743 for the clean_and_jerk, as reported in Figure 7, rather than the 0.2154 that we obtain without a balanced training. Overall, the batch balancing improves the MAE averaged over all categories from 0.1151 to 0.1094.

Overall MAE results are reported in Table 5, where Mean Absolute Error of the architecture is shown in comparison with its variants using oracle joints or class labels. It is clear that the addition of the classification module causes a slight drop in performance of about 3%, as ground truth class labels are no longer used. We also report a static 50% progress baseline for reference.

**Figure 8:**
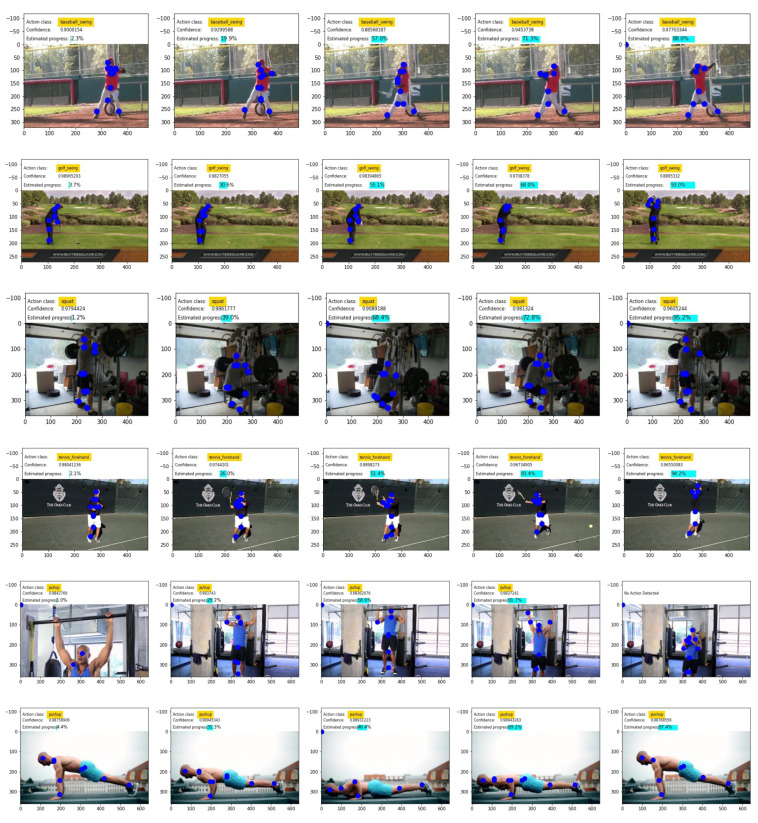
Some examples of the outputs produced by the overall architecture. The first 4 videos are taken from Penn Action dataset whilst the last 2 are taken from YouTube at https://www.youtube.com/watch?v=UgKaDSA3uIg (accessed on 29 December 2022) and at https://www.youtube.com/watch?v=IODxDxX7oi4 (accessed on 29 December 2022).

In addition, in Figure 8, we show some example outputs when videos from the Penn Action dataset are processed by the overall architecture. Here joints are estimated using UniPose and the action is predicted with the action classifier. As can be seen in the figure, as the actions advance, the model is able to correctly estimate their ongoing progress. For each reported frame we show the predicted joints, the predicted class with the confidence of the classifier and the estimated progress. In Figure 8, we also show a few qualitative samples obtained processing videos from YouTube to show the generalization capabilities of the model to unseen videos that are out of the distribution of the Penn Action dataset.

### 5.5. Cyclic Actions

Actions such as push_up, jumping_jacks and other bodyweight exercises are very likely to be performed more than once in a row when it comes to real-life videos. As it is, our model was not trained to cope with cyclic scenarios, thus, when the first repetition ends, the progress prediction will remain stuck at around 95% for all the following ones. The problem stems from the fact that the recurrent neural network of the action progress prediction module will end in a stationary state once the action has reached the end. In order to make the model work in presence of cyclic actions without retraining the model, it would be necessary to identify the end of the action and re-initialize the hidden state of the model to start predicting correctly again. This can be easily achieved by setting a threshold to the predicted action progress: when the esteem is over 95% we reset the model. As a result, it will be ready to evaluate the next action without any previous bias. On the other hand, retraining the model for cyclic actions would solve the issue by instructing the recurrent model to automatically reset the state. If such cyclic actions are not available, data augmentation techniques could be performed in order to generate artificial cyclic actions by repeating of concatenating samples. This could allow a better training of the model, making it able to cope with such situations without the usage of heuristics.

## 6. Conclusions

In this paper we have proposed an approach for estimating action progress in videos based on human poses. Human poses are encoded as body joints, which can be easily extracted by models such as UniPose. We build our model leveraging gated recurrent units to capture temporal dynamics of actions which can be extracted by processing sequences of body poses. The resulting model is extremely lightweight, being able to work in real time, even when estimating joints directly from each frame, as the video is observed. In particular, the action progress estimation module alone can run at more than 1500 FPS on a GPU and can still run at approximately 23 FPS when combining it with UniPose and the action classification module. We tested our model on the Penn Action Dataset, obtaining different levels of prediction accuracy depending on how much precise information can be accessed by the model. However, the model can still obtain low Mean Absolute Errors even when estimating both joints and action categories directly from raw pixels. As for future developments, we intend to merge UniPose and the action classifier in a multi-task fashion: the ResNet backbone module used in UniPose could behave as a feature extractor for the classifier as well. The resulting architecture would be more efficient in terms of resources, and could benefit from information sharing in the multi-task setting.

## Figures and Tables

**Table 1:** Mean absolute error of progress predictions over the test set of the various configurations tested for our model. Best result in bold.

Paradigm	Class Data Available	#GRU Stages	MAE
Many-to-One	✗	1	8.65
Many-to-One	✓	1	6.91
Many-to-One	✓	2	7.05
Many-to-Many	✓	1	6.22
Many-to-Many	✓	2	6.39

**Table 2:** Inference time of the oracle model when processing the 978 test videos of the Penn Action dataset (72,342 frames in total).

	Computing Unit	Total Execution Time	Average Inference Time
**CPU**	Intel Xeon CPU @2.20GHz	69.24 s	1044.86 FPS
**GPU**	NVIDIA Tesla T4	43.77 s	1652.82 FPS

**Table 3:** Mean Absolute Error of progress predictions over the test set of Penn Action dataset when finetuning of the various modules is applied. The last line represents the model with oracle joints. Best result in bold.

Joint Source	UniPose Finetuning	Progress Finetuning	MAE
Unipose	✗	✗	19.82
	✓	✗	11.51
	✓	✓	**7.90**
Oracle	✗	✗	6.22

**Table 4:** Accuracy and Cross-Entropy loss of the tested classifiers over Penn Action Dataset test set. When finetuning is not used, we simply use the model as a feature extraction and train an action classifier on top of the features. The GRU layer instead accumulates information over time and emits a classification at every timestep. Best result in bold.

Backbone	Finetuning	GRU Layer	Test Accuracy
VGG16	✓	✗	70.94
InceptionV3	✗	✗	72.21
InceptionV3	✓	✗	77.23
InceptionV3	✓	✓	**83.04**

**Table 5:** Mean absolute error of progress predictions over the test set of Penn Action dataset of the complete architecture, compared to partial ones and to the constant prediction of 50%. The last column is referred to the scenario in which clean_and_jerk is ignored.

	50% Constant Prediction	Joint-Based Progress Prediction		
Joint source	-	Oracle	Unipose	Unipose
Class source	-	Oracle	Oracle	Classifier
MAE	25.00%	6.22%	7.90%	10.94%

## Data Availability

Publicly available datasets were analyzed in this study. This data can be found here: http://dreamdragon.github.io/PennAction/ (accessed on 29 December 2022).
